# Occupational well-being of diplomatic personnel: a qualitative study

**DOI:** 10.1093/occmed/kqae096

**Published:** 2024-10-11

**Authors:** S K Brooks, D Patel, N Greenberg

**Affiliations:** Department of Psychological Medicine, Weston Education Centre, King’s College London, London SE5 9RJ, UK; Overseas Health and Welfare, Foreign, Commonwealth and Development Office, London SW1A 2AH, UK; Department of Psychological Medicine, Weston Education Centre, King’s College London, London SE5 9RJ, UK

## Abstract

**Background:**

Diplomatic personnel face unique job demands due to their frequent relocations. There is some evidence that occupational stress contributes to poor well-being in this occupational group, but little research on the aspects of the job that are perceived to be particularly challenging.

**Aims:**

This study aimed to explore diplomatic personnel’s perceptions of their organization and their roles and to identify aspects of the job, which could cause stress and potentially affect well-being.

**Methods:**

Semi-structured interviews were carried out with 24 employees of the Foreign, Commonwealth and Development Office between September 2021 and February 2022. Participants were asked to reflect on their experiences before the coronavirus disease 2019 pandemic. Thematic analysis was used to analyse data.

**Results:**

Participants enjoyed many aspects of their work including the variety, travel opportunities and feeling that they made a difference. They also identified several stressors relating to job demands, overseas postings, family needs, frequent relocation, hardship posts, workplace relationships, (lack of) appreciation and acknowledgement, and organizational culture. Good relationships with colleagues and managers were perceived to be very important.

**Conclusions:**

Findings suggest various ways in which diplomatic organizations can better support their personnel, highlighting workload management; cross-cultural training; providing appropriate support to both accompanying families and unaccompanied staff members; practical and psychological preparation for those in high-risk locations; encouraging positive workplace relationships; good management; increased autonomy and increased recognition for good work.

## Teaser

Interviews with 24 diplomatic personnel identified several occupational stressors, including heavy workload, challenges of overseas and hardship postings, concerns about the well-being of family members, frequent relocation, lack of appreciation, and organizational culture. Good relationships with colleagues and managers were deemed essential.

**Key learning points**

**What is already known about this subject:**

- Previous literature suggests international employees face several stressors including difficulties adjusting to new cultures; facing language barriers and facing difficult decisions about whether to travel unaccompanied or bring family overseas.
- Diplomats are a relatively unique subset of international employees due to their frequent relocations (typically moving countries every few years); several studies focussing on diplomats in high-threat posts suggest that they may be negatively affected by potentially traumatic experiences.
- A recent review focussing on diplomats’ mental health highlighted the lack of research on everyday (i.e. not trauma-specific) stressors experienced by diplomatic personnel; this study aimed to fill this gap in the literature.

**What this study adds:**

- This study uses qualitative methodology to gain a richer understanding of diplomats’ perceptions of their work and perceived workplace stressors.
- We found that while diplomatic personnel all identified positive aspects of their work (predominantly the opportunities to travel; variety of the work and the rewarding feeling of making a difference in the lives of others), all participants also identified a number of stressors.
- Stressors included day-to-day job demands such as heavy workload; challenges of overseas postings such as cultural barriers; challenges associated with travelling accompanied or unaccompanied; frequent relocations; exposure to potentially traumatic situations; lack of acknowledgement and workplace culture; good relationships with colleagues and especially managers were deemed essential.

**What impact this may have on practice or policy:**

- The study’s results indicate there are numerous types of training and skills development, which may be beneficial for diplomatic personnel; for example, equipping staff with skills to manage workloads, cross-cultural training and preparing staff both practically and psychologically for high-threat posts would all be helpful.
- Managers within diplomatic organizations could take steps to improve the occupational well-being of their staff by encouraging positive workplace relationships, allowing staff increased autonomy where appropriate and providing recognition and acknowledgement for good work.

## INTRODUCTION

Personnel who work for diplomacy services overseeing international relations (referred to hereafter as ‘diplomatic personnel’) face unique job demands, which could potentially impact their psychosocial well-being. They relocate frequently, needing to adjust to new cultures with various social, economic and political conditions; potentially facing language barriers and contending with difficult decisions about whether to uproot their families or live abroad alone [1,2]. Much of the research on diplomats’ well-being focuses on staff deployed to high-threat postings, who face exposure to potentially traumatic events, and tends to focus on their physical protection rather than psychosocial well-being [3]. A recent scoping review of research examining the mental health of diplomats [2] found only 15 relevant studies. There was little consensus as to the mental health status of diplomatic personnel or factors affecting their mental health. There is some evidence that job-related stress can have detrimental effects on the well-being of diplomatic personnel [4,5] but there has been little research into which aspects of the job are perceived to be most stressful. Additionally, there appears to be no consensus on how to best support staff mental health across diplomatic organizations [6].

This research aimed to explore diplomatic personnel’s perceptions of their work and identify aspects that were particularly challenging/stressful. The recent scoping review on diplomats’ mental health [2] found that only 3/15 studies employed a qualitative methodology, suggesting that the voices of diplomatic personnel themselves are rarely found in the literature. Therefore, this study used qualitative methods to explore diplomatic personnel’s experiences and perceptions in their own words focussing on how diplomatic personnel’s well-being might be affected by various aspects of their roles.

## METHODS

This study used a semi-structured interview design [7]. Participants were employees of the Foreign, Commonwealth and Development Office (FCDO), a UK government department employing over 17 000 staff across the UK and 280 overseas embassies and high commissions [8]. Approximately 27% of these staff work overseas. Eligible staff had to be aged 18 or over and employed by the FCDO for at least 6 months before the interview. Only those who were actively at work would have received invites, which were sent out as ‘work circulars’; thus staff who were away from work (e.g. long-term sick) would not have received the invites.

The research team compiled an invitation letter containing study details and the research team’s contact details. This was emailed by the FCDO Welfare Team to 100 randomly selected staff meeting the inclusion criteria. Due to a low response rate, two further rounds of invitations were sent to a total of another 100 randomly selected staff. Staff emailed the researchers of their own volition and were sent Study Information Sheets and Consent Forms before interviews were arranged.

The interview guide included central questions related to perceptions of the workplace and stressors both in and outside of the workplace. This was part of a wider study exploring diplomats’ well-being during coronavirus disease 2019 (COVID-19) [9]; for the current paper, participants were specifically asked to describe their pre-COVID perceptions and stressors (see Appendix 1). Interviews were carried out using Microsoft Teams, Zoom or over telephone by the first author between September 2021 and February 2022. Interviews were audio-recorded and transcribed verbatim by the first author.

Data were analysed inductively using Braun and Clarke’s six-stage approach to thematic analysis [10]. This involved (i) re-readings of transcripts for familiarization; (ii) breaking down transcripts into initial codes based on their content; (iii) collating codes into overarching themes; (iv) reviewing themes to ensure they reflected the data; (v) naming themes to capture their content and (vi) choosing quotes to illustrate themes. Although coding was done by one author, themes were discussed with the last author as they emerged. Throughout the process, the researcher reflected on how their own experiences or expectations may have influenced the interviews or their interpretation of the data.

The study was carried out according to the British Psychological Society guidelines [11] and data were processed following the General Data Protection Regulation 2016 [12]. Participation was voluntary, with no coercion or consequences for not taking part; participants gave informed consent before participation. All were reassured of their right to withdraw at any time and of confidentiality. The research was approved by the Psychiatry, Nursing and Midwifery Research Ethics Subcommittee at King’s College London (ethical clearance reference number: HR/DP-20/21-22511).

## RESULTS

After 46 employees contacted the researchers for more information about the study, 25 (54%) ultimately took part in the wider study, of whom 24 had worked for the organization pre-pandemic and therefore provided data for this particular part of the study. Interviews lasted between 23 and 58 minutes (median: 33 minutes 41 seconds). Participants worked in a variety of jobs within the FCDO; these were mostly office-based roles based in major cities and included political roles, corporate services, consular teams, and aid policy and strategy roles. Participant demographics are presented in Table 1.

**Table 1.** Participant characteristics

Demographic category	Results
Gender	13 males (54%), 11 females (46%)
Age	Mean 45y; range: early 1920s to mid 1960s
Overseas deployment during career	100%
Countries based in during the past year	*n* = 23 countries across *n* = 6 continents, including 17 ‘hardship locations’ (locations with extremely difficult living conditions) and 2 fragile state posts (locations with substantially impaired economic or social status)
Partner and/or child(ren) living overseas with them	*n* = 15 (63%)
Length of time in role	Mean 14y 8m, median 13.5y (range <2y—30+y)

Thematic analysis identified nine themes: positive aspects of the job; day-to-day job demands; challenges of overseas postings; challenges of both accompanied and unaccompanied posts; challenges associated with moving to new posts; challenges and needs of those in hardship posts; workplace relationships; the importance of acknowledgement and appreciation and organizational culture (see Table 1, available as Supplementary data at *Occupational Medicine* online).

Positively, all participants identified aspects of their roles that they enjoyed (Theme 1: ‘Positive aspects of the job’): most commonly, the variety of tasks involved and opportunities to travel overseas, visit new places, experience new cultures and meet new people (e.g. ‘I do love working overseas’, P2). Participants reported feeling passionate about their work and many described their work as rewarding, and the sense they were doing something worthwhile and making a difference to other people provided them with a sense of achievement.

A number of job demands were described (Theme 2: ‘Day-to-day job demands’). Most participants perceived a high workload, with long hours and short deadlines; this was perceived as stressful and sometimes affected participants’ lives outside of work (e.g. ‘the big stress is always balancing the personal and the professional’, P4). Many reported struggling to find an acceptable work-life balance; they often felt unable to say no or did not want to let others down. Participants reported the amount of administrative work, paperwork and bureaucracy required reportedly caused stress. This, combined with a heavy workload, led to feelings of having too much to do in too little time.

Although overseas postings were highly valued and participants enjoyed experiencing new cultures, they could also be challenging (Theme 3: ‘Challenges of overseas postings’). Being overseas was described as an intense experience that amplified stressors. Living on compounds blurred the boundaries between work and personal life. Those who did not live on compounds felt somewhat isolated from colleagues (e.g. ‘it can be a bit of an ‘us and them’’, P12), which made it hard to form relationships. Language barriers were frequently described as stressful. There were also cultural barriers within English-speaking countries, with different customs and cultures often making it difficult for staff to settle in, assimilate and communicate easily with the local population.

Participants whose close family were with them often faced difficult decisions around partners giving up their careers to accompany them; this could also impact their finances (Theme 4: ‘Challenges of both accompanied and unaccompanied posts’). The lack of stability, and moving every few years, hindered partners finding work or establishing new careers (e.g. ‘[spouses] can’t get a career, that’s always been the biggest problem’, P24). Some participants felt their partners struggled to understand their new roles as homemakers. Others reported their partners having difficulties finding work due to language barriers, difficulties accessing work permits and the lack of work and training opportunities provided by the organization. Additionally, several participants did not feel their partners’ sacrifices were adequately valued by the organization. Other deployed families’ difficulties included leaving support networks and, for children, leaving schools and having to adjust to new ones. Overall, seeing their close family struggling with the adjustment process was perceived to be extremely stressful. Conversely, single/unaccompanied officers described the stress of ‘not’ having family with them; they felt they lacked support and perceived their employer insufficiently considered the needs of those who travelled unaccompanied.

Participants described many challenges associated with moving between posts every few years (Theme 5: ‘Challenges associated with moving to new posts’). Insufficient information about posts, and an application process perceived as arduous, could make finding new posts difficult. Finding posts could be particularly difficult for staff with specific circumstances—such as needing to be in countries where they could access specific medication, or countries with schools which could cater for children with special educational needs. Returning to the UK was also described as difficult, with participants describing struggling with the transition back to living in the UK (e.g. ‘it’s like starting your life again’, P17) and a perceived lack of sufficient support from the organization regarding their return.

Being posted to conflict zones or dangerous locations was reported to be a stressor for many participants; many described being anxious about themselves and their families potentially being in danger (Theme 6: ‘Challenges and needs of those in hardship posts’). Exposure to traumatic situations—including cyclones, hurricanes, mass protests, violence, embassy attacks, military coups, bombings and shootings—reportedly took an ‘emotional toll’ (P15). Multiple exposure to potentially traumatic situations appeared to have a cumulative effect on well-being. Having time and space to decompress after traumatic events was perceived to be helpful. Training and preparation before deployment to hardship posts was deemed essential; while some praised the practical training they received in terms of personal safety, others felt they had not received enough. Those who had previously worked in difficult situations felt they lacked ‘top-up’ training and perceived that the organization assumed they already knew everything. Most participants described feeling psychologically unprepared due to a lack of training in resilience or coping with the traumatic experiences they might face.

Relationships at work appeared to be central to enjoyment of work (Theme 7: ‘Workplace relationships’). When relationships with colleagues (especially managers) were poor, this was perceived to affect well-being and diminish enjoyment of the job. Teams in which colleagues were caring and ‘checked in on’ each other were viewed positively allowing participants to feel they could talk to co-workers easily. Managers were viewed positively if they were caring, supportive and understanding. Participants particularly valued line managers who they could easily talk to about issues and concerns and who allowed them autonomy at work. A minority reported negative relationships with line managers, citing reasons such as poor leadership style, feeling undermined and not being allowed sufficient autonomy.

Acknowledgement and appreciation were described as important (Theme 8: ‘Importance of acknowledgement and appreciation’), but most described feeling insufficiently valued by the organization; this caused ‘a sense of stress and frustration’ (P5).

The organizational culture within the FCDO was frequently described negatively (Theme 9: ‘Organizational culture’): participants described a culture of long hours and working through adversity, with perceived stigma around not being able to cope, and perceived lack of empathy from seniors. However, many participants also perceived the culture to be evolving and improving due to changes in leadership and subsequent changes in culture.

## DISCUSSION

All participants reported enjoying some aspects of their work particularly opportunities to live overseas, the breadth and variety of tasks and feeling their work made a difference. However, work challenges were frequently described, including heavy workload; cultural and language barriers when based overseas; challenges specific to hardship posts; challenges of frequent relocation and negative aspects of organizational culture. This study supports previous research in terms of the stressful effects of having to adapt to working in new countries and cultures [13] and the potential negative psychological effects of workplace trauma exposure [14,15].

As stated, the interviewer reflected on how their own expectations may have influenced data analysis. For example, they had previous experience of conducting research with other governmental departments. However, they consciously questioned their assumptions and encouraged participants to talk freely about their lived experiences. There are limitations to the current study in terms of the relatively small sample size, the fact that only 12% of those contacted and 54% of those who requested information took part in the study, the potential for social desirability bias in participants’ responses, and the fact that participants were asked to retrospectively recall pre-COVID stressors at a time when the pandemic was ongoing. The latter point potentially affects the validity of the results due to the possibility of recall bias or of the current, likely stressful COVID-19 situation causing participants to recall experiences more negatively. Nevertheless, the study makes an important contribution to the small body of literature examining the occupational well-being of diplomatic personnel. To our knowledge, this is the first qualitative study to examine the day-to-day (i.e. not specifically trauma-related) stressors experienced by diplomatic personnel, identifying several aspects of the work that were perceived as stressful.

Long hours, heavy workloads and excessive administrative and bureaucratic tasks often led to evenings, and weekend working to meet deadlines, making it difficult to find an appropriate work-life balance. It may thus be that providing ‘workload management’/prioritization training for employees, which focuses on how to prioritize work and when to delegate [16] could enhance staff’s skills for coping with heavy workloads.

Additionally, both language and cultural barriers made work more difficult, hindering staff who were settling into new posts and integrating with the local communities. Additional cross-cultural training and education focussed on adjusting to new cultures, may help develop cultural understanding, enhance ability to communicate and develop positive relationships and thus help staff adjust more rapidly [17]. Bringing families overseas often involves partners having to give up their careers, which could harm their financial situation as well as their partners’ mental health. Staff often felt their employer did not fully appreciate their family’s sacrifices. This finding echo research on the romantic partners of military personnel [18]. Meanwhile, single or unaccompanied officers felt lacking of support or recognition of how difficult it was to take care of homes and administrative work by themselves, on top of full-time jobs. It may be beneficial for diplomatic organizations to provide greater training and employment opportunities for employees’ accompanying partners and ensure that line managers regularly check in on single/unaccompanied officers to ensure they are not overlooked in terms of support.

Many participants had previously been exposed to potentially traumatic situations, which appeared to have taken a psychological toll. Whilst most staff had received practical safety training, more trauma-experienced staff felt it was simply assumed that they already knew what to do, whilst others felt their families did not receive adequate training. Diplomatic organizations should consider taking a more systematic approach to training [6], ensuring that all staff (and their families) deployed to conflict zones receive an appropriate training. Additionally, participants reported having little preparation for the potential psychological impact of being exposed to such situations, echoing previous research suggesting that greater training for psychological preparation in trauma-exposed organizations is needed [19]. While there is some evidence that pre-exposure training can improve confidence, further research is needed to ascertain what kind of pre-exposure training is most effective [20]. Given the importance of decompression described by the participants, diplomatic organizations (and indeed any trauma-exposed organizations) should ensure the provision of time and space to discuss and make sense of experiences after experiencing potentially traumatic events [21].

Collegial relationships at work were reported as being central to staff’s enjoyment of their jobs. In particular, relationships with line managers appeared to have a strong influence on how they perceived their jobs. This is very much in keeping with prior research findings in non-diplomatic organizations [21,22]. This finding strongly suggests that personnel in supervisory positions should be trained in how to effectively manage their employees including being clear in their directions and feedback; dealing with problems as they arise and supporting staff appropriately through difficult situations. Training and workshops designed to enhance leadership skills are likely to be beneficial [21,22]. Workplace relationships with colleagues were also important to our participants. Research suggests that strong social networks promote resilience [23]. Finding ways to enhance team cohesion, such as through team events, is likely to be useful.

Our findings also highlight the importance of job autonomy. Poor control and autonomy at work have been associated with poor mental health in many occupational groups [24,25]. Strategies for enhancing autonomy include allowing participation in decision-making; including employees on organizational committees; having specific workgroups promoting employee involvement in decision-making and enhancing competence in decision-making through training and education [26].

Many participants reported feeling undervalued. Research with other occupational groups suggests this can have a substantial negative impact on staff well-being [27,28]. Employees who do feel valued experience greater job satisfaction, happiness, productivity and commitment to their organizations [29]. Our finding is consistent with the Effort-Reward Imbalance model of occupational stress [30] which suggests efforts required to meet job demands should be balanced by rewards, either in terms of pay, career opportunities, esteem, positive feedback or feeling valued. Line managers could address the perceived effort-reward imbalance in several ways, such as by providing positive feedback and acknowledgement; promoting staff development; setting up award programmes to award good performance and performance evaluation meetings [31].

Further research on the occupational well-being of diplomatic personnel is warranted. Additionally, further research on other organizations deploying employees internationally could help diplomatic organizations understand how other relevant organizations experience overseas working.

To conclude, to our knowledge this is the first qualitative study exploring diplomatic personnel’s perceptions of the workplace. Our findings identify several distinct workplace stressors and suggest that various types of training might be beneficial (e.g. workload management training, cross-cultural training, training in how to psychologically prepare for traumatic exposure). Findings also highlight the importance of managers being good leaders and identify ways in which they can support their staff (e.g. fostering positive relationships within teams, increasing autonomy, acknowledgement of good work).

## Supplementary Material

kqae096_Supplementary_Table_1
