# Supplementary material for: Occupational well-being of diplomatic personnel: a qualitative study
Source: Occup Med (Lond). 2024 Oct 11;75(6):313–8. doi: 10.1093/occmed/kqae096 (PMC12449242; doi:10.1093/occmed/kqae096)
Supplement: kqae096_Supplementary_Table_1 [file kqae096_supplementary_table_1.docx]

**Supplementary Table 1. List of themes, sub-themes and quotes illustrating each sub-theme**

| Theme | Sub-theme | Quotes |
| --- | --- | --- |
| 1. Positive aspects of the job | Variety | “[I like] being able to change my job every three to four years (…) the job is never the same in different countries” (P17); “It’s wonderful and a privilege to be able to work on a very wide variety of things” (P12); “I like the variety, I like the fact that you do change roles every couple of years or so” (P21) |
|  | Opportunities to live and work overseas | “I enjoy the travel (…) I enjoy the people, the locals” (P6); “Genuinely love being on overseas postings (…) learning about new cultures and countries” (P19); “I do love working overseas, that’s part of why I joined the Foreign Office in the first place” (P2) |
|  | Passionate staff | “I think one of the brilliant bits about working there (…) is that most of the staff you work with are passionately interested in what they do, and they care, they really care about what they do and that’s a wonderful thing to work with” (P12) |
|  | ‘Making a difference’ | “I love (…) doing just really worthwhile work” (P19); “Even when you’re really busy, you get an adrenaline rush because you feel you’re making a difference” (P5) |
| 2. Day-to-day job demands | Workload | “This massively increasing workload relative to the numbers of staff” (P12); “Too much work and not enough time” (P21); “Workload, sheer volume of work, and some (…) uncompromising deadlines from ministers” (P1) |
|  | Work-life balance | “You can’t step away from work, like even if I am spending an evening not working, I know very much that my ambassador or seniors could come and ask me to work (…) that kind of expectation does make it quite difficult to fully switch off” (P3); “The big stress is always balancing the personal and professional there, because the job is very demanding (…) getting that balance right so that you have enough time for your family, it’s really important and can be quite challenging” (P4) |
|  | Bureaucracy | “It’s very bureaucratic (…) there's always forms you have to fill in, there's always policies that quite often make no sense” (P5); “It’s all the admin that you have to go through where you don’t really get much support” (P13) |
|  | Intellectually demanding work | “There’s always a lot happening politically, which we have to follow, engage with” (P4); “[Work is] sometimes quite difficult, intellectually – you know you’re working with very, very bright people on some very important issues” (P19) |
| 3. Challenges of overseas postings | Intensity of being overseas | “The hard thing about being overseas is the intensity (…) the intensity gets really dialled up” (P12); “It’s become such an enormous part of your life in a way that it doesn’t when in London you leave the office at five o’clock and that’s it, you’re out in the world again” (P12) |
|  | Living situation | “You came home from work and you went onto your balcony and you just stared thirty foot across the courtyard to your colleague maybe you had a difficult day at work with. And that can be really difficult” (P12); “We’re spread so far and wide (…) the sense of community certainly within my team and the sense of community within the embassy is just not there” (P10) |
|  | ‘Us and them’ divisions | “There’s like two different communities here. There’s the locally based staff who have that right way of doing things, and then there’s those of us that dip in for a couple of years (…) and then dip out again” (P10); “A lot of the local staff work in the embassy for many years and so it can be a bit of an ‘us and them’, and it can be very hard to [make] friendships” (P12) |
|  | Language and cultural barriers | “The big thing is, the major challenge we face (…) is the language barrier is quite a big one” (P7); “People under-estimate the complexities of moving to [country] because whilst we do speak the same language (…) there’s so many fundamental differences, there are cultural differences” (P24); “I think there’s also the perception that (…) because they speak a similar language that it’s like being at home, and it’s not” (P10) |
|  | Missing family and friends at home | “Just not having your family nearby, who we can say [to] we're having a really rough time (…) that was probably the biggest stress for us outside of work” (P17); “Even if you’ve (…) made friends in your postings, they’re not friends and family who you’ve known for twenty to thirty years” (P16) |
| 4. Challenges of both accompanied and unaccompanied posts | Accompanying partners giving up work | “We would be a couple with a child with only one income, whereas had I never gone overseas, we would have had a two-income household (…) I’m well aware of the effect on the lifetime earnings of my spouse” (P12); “Spouses overseas, they can often get a job if they’ve got the right work experience, but they can’t get a career, that’s always been the biggest problem” (P22); “What's also been hard is she's worked the whole time [in the UK] (…) and then coming out here I think it's been really difficult for her to understand quite what her role is” (P24) |
|  | Accompanying families leaving their support networks | “My wife has particularly found it hard because she's close to her family (…) [I’ve] become slightly desensitized to those farewells. But my wife has never experienced that” (P24); “[Child] finds it difficult to make friends initially because the whole process is just a bit intimidating again and again” (P24) |
|  | (Perceived) lack of organisational support for single / unaccompanied officers | “We spend a lot of money and time and effort on accompanied officers, I think we need to spend a bit more time focusing on single officers (…) kind of reviewing what we do for single officers overseas” (P16) |
| 5. Challenges associated with moving to new posts | Challenges of frequent relocations | “It’s exciting isn’t it, to work in different places, but it’s also on the flip side quite exhausting moving around” (P12); “No matter how much you really research which post to choose, the material available to give you a feeling for what a post might be like never really describes the situation. You don’t know until you get there” (P23) |
|  | Challenges of returning to the UK | “It's like starting your life again (…) ‘oh, you're home, you know what you're doing’ but (…) two or three years overseas, then boom, you’re going home all of a sudden, it's actually more terrifying than going (…) somewhere else” (P17) |
| 6. Challenges and needs of those in hardship posts | Fears for safety | “[Country]’s got high crime so you don’t go out at night because that’s when the attacks really happen, so that puts this bit of stress on you and the family” (P6); “If you Google [country] and you look at photos it all looks very lovely and it is very lovely for a (…) holiday, but they can be difficult places to live” (P12) |
|  | Exposure to potentially traumatic experiences | “Just constantly being exposed to all these different kinds of things, that takes its toll (…) I need to stop working, I need to step off because I can’t continue anymore because of the impact of this, psychological impact of all those different things just adding up over time” (P15); “I can be called out to any crisis at any time (…) the body needs to recover, the head needs to recover. You’re working twenty-hour days helping people. It becomes stressful on the mind and the body” (P6) |
|  | Importance of decompression | “I was able to take three weeks off as a block and that just made a huge difference” (P15); “You work really intensely for those six weeks and then for two weeks you go home and the minute you’re on that plane you can just kind of forget everything and it’s a proper break” (P16) |
|  | Importance of practical training and preparation | “They prepare you for what gunfire sounds like and how to react at checkpoints and those kind of quite practical things, which I think is really important (…) it familiarises you with those things that would be otherwise quite unfamiliar, and I think that makes them far less daunting and scary, which I think definitely helps” (P3); “I’d say potentially, if I hadn’t been in crisis situations before, it might have been a little bit of a shock arriving” (P16); “The actual training itself is very limited, you may get some job-shadowing (…) where you can then learn what the job entails and how to do it but it doesn’t happen for everybody (…) there’s an assumption that you sort of already know how to deal with it” (P15) |
|  | Importance of psychological training and preparation | “In terms of actual formalised training and how you would deal with that (…) there’s nothing that was given to us” (P7); “There isn’t really kind of any preparation I found for that more kind of resilience aspect to it, that kind of day-to-day living and working in a post like this” (P3) |
| 7. Workplace relationships | Importance of good relationships with colleagues | “A really big part of how you feel about your work environment and your work situation and what you are doing as a person in terms of your own personal sense of fulfilment comes down to the colleagues that you’re working with” (P23); “The biggest stress factor would be if I’d been in teams where there wasn’t a very supportive environment” (P16); “Another source of stress would be if there’s internal conflicts between colleagues” (P5) |
|  | Qualities of a good team | “It’s mutually supportive (…) [we] take care of each other, make sure that things are going well, because the nature of our jobs cross over a lot of the time” (P15); “You know if you are struggling a bit they just kind of go right, you just need to step away and we’ll field anything that comes through” (P13); “They are very caring, people really care about one another (…) it’s a very caring inclusive office in general” (P3) |
|  | Importance of good relationships with managers | “Most of the time you’ve got a good team, but the impact is more felt if you’ve got clash with management and your direct line manager or the one above” (P21); “I was due to do four years here but I’m [doing less] because I can’t work with people like [manager]” (P6) |
|  | Qualities of a good manager | “I had quite a bit going on for a while and it was my boss who was quite supportive and she was really understanding (…) and if it hadn’t been for her I don’t know who else I would have talked to about anything” (P21); “The interaction I have with senior leaders both on a personal and professional level has always been incredibly caring and supportive, I think that’s something I really love about the Office” (P3); “I’ve just got to say to [manager] look, I’m struggling a bit with this, and she’s there” (P13); “He’s a very good line manager, he knows that I know my job (…) and just lets me get on with my job” (P6) |
|  | Qualities of a poor manager | “Management not being clear on what it is that they’re trying to do” (P21); “Making people managers without training them to be a manager” (P12); “He was quite negative, he just was basically looking for his next job and it soon became apparent (…) he had no interest in being a line manager” (P2); “I don’t need micro-managing (…) you just gotta let us do our job properly instead of undermining us (…) you get very demoralised when you've got (…) people telling you how to do your job or telling you you can’t do your job” (P6) |
| 8. Importance of acknowledgement and appreciation | Importance of feeling valued | “I think sometimes as an organisation they don’t always take that time to just step back and say, ‘actually, you’re doing an amazing job, everybody here is doing an amazing job’ (…) just to say you know, ‘you are doing an amazing job’, and use language like that, and say ‘thank you (…) I really do appreciate it’. And it being meant. I think this is hugely important” (P13) |
|  | Lack of appreciation / reward | “I don’t think we are recognised as much as we could be. We in our team tend to be the team that people go to when all else has failed, they come to us and we end up being the sort of punchbag for the rest of the office (…) we don’t feel appreciated” (P11); “There are certain key skills in the organisation that we need to retain (…) being good line managers, coaches, mentors, supporters of colleagues in general, that’s not something that’s rewarded, it’s something they say that’s important, but in terms of the kind of reward and pay structures, whether that’s appraisals, promotions et cetera, that’s not something that’s tied to that reward structure” (P2); “I think that can cause a sense of stress and frustration, if you feel that you’re being marginalised, you’re not being valued, your work’s not valued and it’s not even recognised as vaguely important” (P5) |
| 9. Organisational culture | Lack of empathy | “There are some very senior people who maybe have been around a long time and empathy is not their main focus” (P16); “I think [the organisation] needs to be more trusting and kinder” (P5); “I think they need to be able to indicate that they empathise with people” (P12) |
|  | Culture of long hours and working through adversity | “There’s a certain culture I think in the Office that you should come in and every day be kind of happy and optimistic and extroverted and outgoing, and (…) that is a very, very, very high bar” (P12); “It was very much, you have to be in the office, it’s a long hours culture where we all know being in the office for long hours doesn’t mean you’re working any more successfully than someone who’s able to cram in loads of work in set periods of time. (…) That’s the kind of thing that needs to change” (P5) |
|  | Perceived stigma around difficulties coping | “I thought that [if] I really wasn't coping at the moment I could just say to [my line manager], you know I've got to take a bit of time out, I'm not coping (…) but it’s totally against the culture in the Office” (P11) |
|  | Evolving culture | “I think I have a sense that their culture is changing. It seems so much more kind than it used to be (…) I think the Foreign Office is catching up and changing that culture” (P5); “We just have to take control of our own welfare a lot of the time (…) that's what they should be doing, the whole kindness thing, be kind to each other. And that's the sort of culture that is being fostered now (…) I just think it will take a long time” (P11) |
